# Additive Effect of Physical Activity and Sedentary Time on Depressive Symptoms in Rural Japanese Adults: A Cross-Sectional Study

**DOI:** 10.2188/jea.JE20180017

**Published:** 2019-06-05

**Authors:** Takafumi Abe, Tsuyoshi Hamano, Keiichi Onoda, Miwako Takeda, Kenta Okuyama, Masayuki Yamasaki, Minoru Isomura, Toru Nabika

**Affiliations:** 1 Center for Community-Based Healthcare Research and Education (CoHRE), Organization for Research and Academic Information, Shimane University, Shimane, Japan; 2 Department of Sports Sociology and Health Sciences, Faculty of Sociology, Kyoto Sangyo University, Kyoto, Japan; 3 Department of Neurology, Faculty of Medicine, Shimane University, Shimane, Japan; 4 Faculty of Human Sciences, Shimane University, Shimane, Japan; 5 Department of Functional Pathology, Faculty of Medicine, Shimane University, Shimane, Japan

**Keywords:** physical activity, sedentary behavior, depressive symptoms, community, public health

## Abstract

**Background:**

Previous studies have reported an additive effect of moderate-to-vigorous physical activity (MVPA) and sedentary time (ST) on depressive symptoms. However, no studies have been conducted in rural community settings. This cross-sectional study investigated whether the additive effect of MVPA and ST was associated with depressive symptoms in rural Japanese adults.

**Methods:**

We identified 2,814 participants from health examinations conducted in Shimane, rural Japan, in 2012 and analyzed data from 1,958 participants. We assessed depressive symptoms using the Zung Self-Rating Depression Scale and measured the total time spent on MVPA and ST using a Japanese short version of the International Physical Activity Questionnaire. Poisson regression analysis examined the prevalence ratios (PR) of depressive symptoms in nine category combinations of MVPA level (no, insufficient, or sufficient MVPA) and ST level (high, moderate, or low ST).

**Results:**

A total of 117 (6.0%) participants had depressive symptoms. Compared with the reference category (no MVPA/high ST), multivariate analysis showed that the likelihood of depressive symptoms was significantly lower in the sufficient MVPA/low ST category (PR 0.23; 95% confidence intervals [CI], 0.08–0.66), insufficient MVPA/low ST category (PR 0.37; 95% CI, 0.16–0.86), and insufficient MVPA/moderate ST category (PR 0.39; 95% CI, 0.17–0.90).

**Conclusion:**

Analysis of the additive effect of MVPA and ST showed that the combinational category of sufficient MVPA and low ST had the lowest prevalence of depressive symptoms in rural Japanese adults. Moderate ST and low ST showed significantly lower likelihoods of depressive symptoms, regardless of insufficient MVPA.

## INTRODUCTION

Depression was the third leading contributor to the global burden of disease in 2004 and is projected to be the leading contributor globally in 2030.^1^ The prevalence of depression has increased in the United States during the last 2 decades.^2^^,^^3^ In Japan, the number of patients with mood disorders, including depression, has increased from 433,000 in 1996 to 1,116,000 in 2014.^4^ Depression is a known risk factor for cardiovascular disease,^5^ stroke,^6^ type 2 diabetes,^7^ and mortality.^8^ Therefore, preventing depressive symptoms is important within the field of public health.

Several risk factors for depressive symptoms have been identified, including socioeconomic factors,^9^ poor health conditions,^10^ and lifestyle factors.^11^ Previous reviews have shown that physical activity (PA) is independently associated with the onset of depressive symptoms.^12^^,^^13^ Sedentary behavior (SB), which is related to PA, has been a subject of great interest. SB refers to any waking behavior characterized by an energy expenditure of ≤1.5 metabolic equivalents, while in a sitting, reclining, or lying posture.^14^ High levels of sedentary time (ST) have been related detrimentally to cardiovascular disease, type 2 diabetes, and cardiovascular and all-cause mortality.^15^^,^^16^ A recent meta-analysis has also demonstrated that ST is significantly associated with an increased risk of depression.^12^

In a 20-country survey, Japan had the highest prevalence of self-reported ST.^17^ Owing to the difference in street layout between rural and urban areas in Japan, rural residents were less likely to meet the recommended levels of PA than urban residents, and TV watching time tended to be longer.^18^ Although a few studies have reported an additive effect of MVPA and ST on depressive symptoms in Japan,^19^ to the best of our knowledge, no studies have been conducted in a rural community setting. We hypothesized that rural residents meeting the recommended levels of MVPA with lower ST are less likely to have depressive symptoms than those not meeting the recommended levels of MVPA with high ST. Therefore, the present study aimed to examine whether there was an additive effect of MVPA and ST on the onset of depressive symptoms in rural Japanese adults.

## METHODS

### Participants

This cross-sectional study was part of a cohort study conducted by the Center for Community-Based Healthcare Research and Education, Shimane University (Shimane CoHRE Study). The study protocol was approved by the Ethics Committee of Shimane University (#2888), and written informed consent was obtained from all participants prior to enrolment. The study procedure, analysis, and description are reported according to the Strengthening the Reporting of Observational Studies in Epidemiology (STROBE) statement.^20^

**Figure 1.**
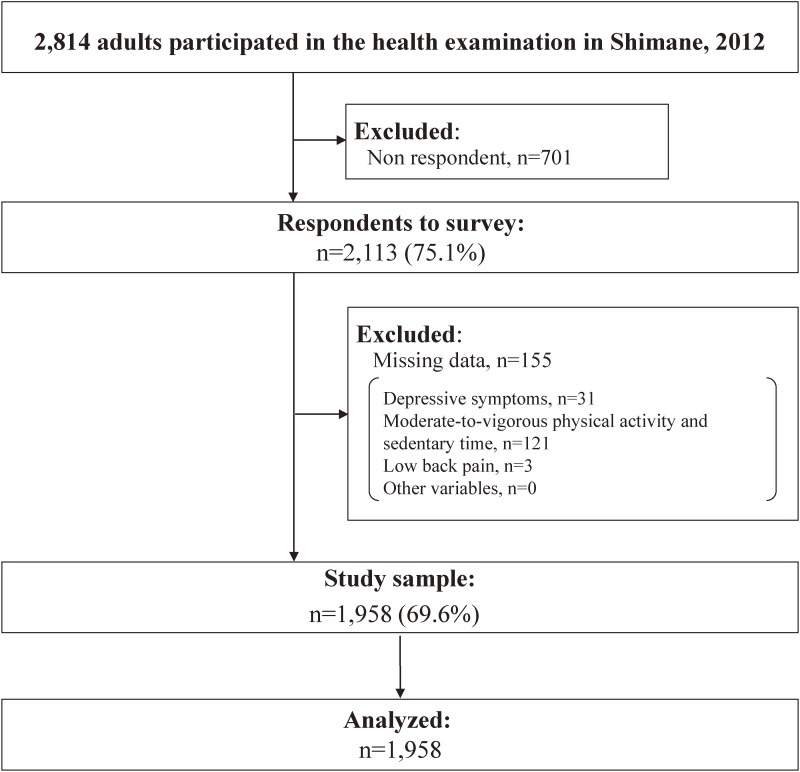
Flowchart of the study

Data were collected from health examinations conducted in Shimane, rural Japan, from June through November 2012. In all, 2,814 adults participated in the health examinations. After excluding participants with non-responses (*n* = 701) and 155 participants with missing data (depressive symptoms, *n* = 31; MVPA and ST, *n* = 121; low back pain, *n* = 3; other variables, *n* = 0), we analyzed data from 1,958 participants (Figure 1).

### Outcome variable: depressive symptoms

The outcome variable was the presence of depressive symptoms, assessed using the Zung Self-Rating Depression Scale (SDS), a 20-item self-report questionnaire.^21^ Each item is scored from 1 to 4, and the total score ranges from 20 to 80; higher scores indicate more severe depressive symptoms. A Japanese version of the SDS has been developed.^22^ The test-retest reliability over 7 days for each item was acceptable (*r* > 0.60). In the validation study, a chi-squared analysis showed a statistically significant difference in scores on each item between healthy people and depressive patients (*P* < 0.05). Moreover, the total SDS score of depressive patients was significantly higher than that of healthy people (*P* < 0.01). In this study, a cut-off point (SDS score ≥48) was used to define depressive symptoms.

### Exposure variables: physical activity and sedentary behavior

MVPA and ST were evaluated using the short version of the International Physical Activity Questionnaire (IPAQ-SV).^23^ The test-retest reliability and criterion validity of the Japanese version of the IPAQ-SV have been confirmed.^24^ The total time spent on MVPA was calculated from participant reports of the frequency and duration of three types of PA (vigorous intensity, moderate intensity without walking, and walking). The total time spent on MVPA was divided into three categories using cut-off values from previous studies.^19^ The recommended MVPA level was defined as “sufficient MVPA” (≥150 min/week). The thresholds were based on the current PA recommendations.^14^^,^^26^^–^^28^ Physical inactivity was categorized as “insufficient MVPA” (1 to <150 min/week), or “no MVPA” (0 min/week). ST was estimated by the total time spent on SB per day as follows: (ST on weekdays × 5 + ST on weekends × 2)/7, and divided into three categories: “low ST” (<3 h/day), “moderate ST” (3 to <6 h/day), or “high ST” (≥6 h/day) using cut-off values from previous studies.^19^^,^^25^

### Other variables

Sex (male or female), age (years, divided as a categorical variable), residential area (city of Unnan, town of Okinoshima, or town of Ohnan), having enough sleep (yes or no), current alcohol drinking (yes or no), current smoking (yes or no), having low back pain (yes or no), history of heart disease (yes or no), and history of stroke (yes or no) were ascertained using a face-to-face interview conducted by trained staff. Blood pressure, height, and weight were measured by public health nurses. Blood pressure was divided into two categories using a cut-off point (systolic blood pressure/diastolic blood pressure = 140/90 mm Hg).^29^ Body mass index (BMI) was calculated from measured data of height and weight in kg/m^2^ and divided into three categories using cut-off points (underweight: <18.5; normal: 18.5 to 22.9; overweight: ≥23.0 kg/m^2^).^30^

### Statistical analysis

Descriptive statistics were calculated for the prevalence of all variables according to depressive symptoms. Statistical significance of the differences between groups was determined using a chi-squared test.

In the cross-sectional analyses, multivariable-adjusted Poisson regression analyses were calculated to estimate the prevalence ratios (PRs) and 95% confidence intervals (CIs) for having depressive symptoms in nine joint categories that included both MVPA levels (no, insufficient, or sufficient MVPA) and ST levels (high, moderate, or low ST).^31^ Independent variables were adjusted for sex, age, and BMI in model 1, and model 2 included all variables from model 1 and city/town of residence, having enough sleep, current alcohol drinking, current smoking, low back pain, blood pressure, heart disease history, and stroke history. For the sensitivity analysis, Poisson regression analysis was used to examine depressive symptoms with six combination categories that included both MVPA levels (low MVPA, high MVPA; using the median value of 55.8) and ST levels (high, moderate, and low ST). All statistical analyses were carried out using IBM SPSS Statistics 24.0 for Windows (IBM Corp., Armonk, NY, USA). For all analyses, *P*-values less than 0.05 were considered statistically significant.

## RESULTS

Table 1 shows differences in depressive symptoms according to participant characteristics. Of 1,958 participants, 117 (6.0%) had depressive symptoms. The prevalence of depressive symptoms was significantly different among the no MVPA (10.1%), insufficient MVPA (5.8%), and sufficient MVPA (4.8%) groups (*P* = 0.03). The prevalence of depressive symptoms was also significantly different among the high ST (10.5%), moderate ST (5.7%), and low ST (5.0%) groups (*P* < 0.01). Furthermore, there was a significant difference in the prevalence of depressive symptoms for BMI (*P* < 0.01), city/town of residence (*P* = 0.03), having enough sleep (*P* < 0.001), current smoking (*P* < 0.01), low back pain (*P* < 0.01), and history of heart disease (*P* < 0.01). There was no significant difference in the prevalence of depressive symptoms for sex, age, current alcohol drinking, blood pressure, or history of stroke.

**Table 1.** Characteristics of study participants

Characteristics	Total *N* = 1,958	No depressive symptoms *n* = 1,841 (94.0)	Depressive symptoms^a^ *n* = 117 (6.0)	*P*-value^b^
Physical activity				
No MVPA (0 min/week), *n* (%)	189 (9.7)	170 (89.9)	19 (10.1)	0.03
Insufficient MVPA (1 to <150 min/week), *n* (%)	1328 (67.8)	1251 (94.2)	77 (5.8)	
Sufficient MVPA (≥150 min/week), *n* (%)	441 (22.5)	420 (95.2)	21 (4.8)	
Sedentary time				
High ST (≥6 h/day), *n* (%)	248 (12.7)	222 (89.5)	26 (10.5)	<0.01
Moderate ST (3 to <6 h/day), *n* (%)	883 (45.1)	833 (94.3)	50 (5.7)	
Low ST (<3 h/day), *n* (%)	827 (42.2)	786 (95.0)	41 (5.0)	
Sex				
Male, *n* (%)	758 (38.7)	720 (95.0)	38 (5.0)	0.15
Female, *n* (%)	1200 (61.3)	1121 (93.4)	79 (6.6)	
Age				
59 years and below, *n* (%)	213 (10.9)	192 (90.1)	21 (9.9)	0.09
60–69 years, *n* (%)	737 (37.6)	698 (94.7)	39 (5.3)	
70–79 years, *n* (%)	890 (45.5)	840 (94.4)	50 (5.6)	
80 years and above, *n* (%)	118 (6.0)	111 (94.1)	7 (5.9)	
Body mass index				
Underweight (<18.5 kg/m^2^), *n* (%)	144 (7.4)	127 (88.2)	17 (11.8)	<0.01
Nomal (18.5 to <22.9 kg/m^2^), *n* (%)	1008 (51.5)	960 (95.2)	48 (4.8)	
Overweight (≥23.0 kg/m^2^), *n* (%)	806 (41.2)	754 (94.0)	117 (6.0)	
City/Town of residence				
City of Unnan, *n* (%)	896 (45.8)	856 (95.5)	40 (4.5)	0.03
Town of Okinoshima, *n* (%)	186 (9.5)	173 (93.0)	13 (7.0)	
Town of Ohnan, *n* (%)	876 (44.7)	812 (92.7)	64 (7.3)	
Having enough sleep				
Yes, *n* (%)	1519 (77.6)	1466 (96.5)	53 (3.5)	<0.001
No, *n* (%)	439 (22.4)	375 (85.4)	64 (14.6)	
Current alcohol drinking				
No, *n* (%)	1019 (52.0)	950 (93.2)	69 (6.8)	0.12
Yes, *n* (%)	939 (48.0)	891 (94.9)	48 (5.1)	
Current smoking				
No, *n* (%)	1788 (91.3)	1689 (94.5)	99 (5.5)	<0.01
Yes, *n* (%)	170 (8.7)	152 (89.4)	18 (10.6)	
Low back pain				
No, *n* (%)	1063 (54.3)	1016 (95.6)	44 (4.4)	<0.01
Yes, *n* (%)	895 (45.7)	825 (92.2)	70 (7.8)	
Systolic/Diastolic blood pressure				
<140/90 mm Hg, *n* (%)	1386 (70.8)	1305 (94.2)	81 (5.8)	0.70
≥140/90 mm Hg, *n* (%)	572 (29.2)	536 (93.7)	36 (6.3)	
History of heart disease				
No, *n* (%)	1817 (92.8)	1716 (94.4)	101 (5.6)	<0.01
Yes, *n* (%)	141 (7.2)	125 (88.7)	16 (11.3)	
History of stroke				
No, *n* (%)	1893 (96.7)	1782 (94.1)	111 (5.9)	0.26
Yes, *n* (%)	65 (3.3)	59 (90.8)	6 (9.2)	

MVPA, moderate-to-vigorous physical activity; ST, sedentary time.

^a^The prevalence of depressive symptoms (%).

^b^Differences between depressive symptoms (no or yes) were examined using chi-square tests for categorical variables.

Table 2 shows the joint association of MVPA and ST with the prevalence of depressive symptoms in the Poisson regression. After adjusting for sex, age, and BMI (model 1), the sufficient MVPA/low ST category (PR 0.18; 95% CI, 0.06–0.53) had the lowest likelihood of depressive symptoms compared with the reference category (no MVPA/high ST category), and this difference was significant. In addition, the insufficient MVPA/low ST category (PR 0.26; 95% CI, 0.11–0.61), the insufficient MVPA/moderate ST category (PR 0.29; 95% CI, 0.13–0.66), and the sufficient MVPA/moderate ST category (PR 0.34; 95% CI, 0.13–0.87) were significantly associated with less depressive symptoms compared with the reference category. After adjusting for all confounders (model 2) in the multivariable analysis, the sufficient MVPA/low ST category (PR 0.23; 95% CI, 0.08–0.66) had the lowest likelihood of depressive symptoms compared with the reference category (no MVPA/high ST category), and this difference was significant. The insufficient MVPA/low ST category (PR 0.37; 95% CI, 0.16–0.86) and insufficient MVPA/moderate ST category (PR 0.39; 95% CI, 0.17–0.90) also had a significantly lower likelihood of depressive symptoms compared with the reference category. The other categories were not significantly associated with depressive symptoms. The sensitivity analysis yielded similar results to the main analysis (Table 3). In model 2, the high MVPA/low ST category (PR 0.39; 95% CI, 0.21–0.73) had the lowest likelihood of depressive symptoms compared with the reference category (low MVPA/high ST category), and this difference was significant. The high MVPA/high ST category was not significantly associated with depressive symptoms.

**Table 2.** Joint associations of moderate-to-vigorous physical activity and sedentary time with depressive symptoms: cross-sectional analysis

Variables	Cross-sectional analysis (*N* = 1,958)			

	Sample	Depressive symptoms,^a^ %	PR (95% CI)^b^	

			Model 1^c^	Model 2^d^
No MVPA/High ST	37	18.9	1.0 (reference)	1.0 (reference)
No MVPA/Moderate ST	88	6.8	0.36 (0.12–1.08)	0.45 (0.15–1.36)
No MVPA/Low ST	64	9.4	0.50 (0.17–1.49)	0.52 (0.17–1.60)
Insufficient MVPA/High ST	176	9.7	0.52 (0.21–1.25)	0.58 (0.24–1.42)
Insufficient MVPA/Moderate ST	598	5.4	**0.29 (0.13–0.66)**	**0.39 (0.17–0.90)**
Insufficient MVPA/Low ST	554	5.1	**0.26 (0.11–0.61)**	**0.37 (0.16–0.86)**
Sufficient MVPA/High ST	35	5.7	0.32 (0.07–1.55)	0.36 (0.07–1.75)
Sufficient MVPA/Moderate ST	197	6.1	**0.34 (0.13–0.87)**	0.45 (0.17–1.15)
Sufficient MVPA/Low ST	209	3.3	**0.18 (0.06–0.53)**	**0.23 (0.08–0.66)**

BMI, body mass index; CI, confidence interval; MVPA, moderate-to-vigorous physical activity; PR, prevalence ratio; ST, sedentary time.

The total time spent on MVPA was categorized into “sufficient MVPA” (≥150 min/week), “insufficient MVPA” (1 to <150 min/week), and “no MVPA” (0 min/week). ST classified by three categories: “low ST” (<3 h/day), “moderate ST” (3 to <6 h/day), and “high ST” (≥6 h/day).

^a^Depressive symptoms were dichotomized using cut-off (scores ≥48) on the Zung Self-Rating Depression Scale.

^b^Prevalence ratios and 95% confidence intervals were estimated using Poisson regression.

^c^Adjusted for sex, age, and BMI.

^d^Adjusted for sex, age, BMI, town/city of residence, having enough sleep, current alcohol drinking, current smoking, low back pain, blood pressure, history of heart disease, and history of stroke.

**Table 3.** Joint associations of moderate-to-vigorous physical activity and sedentary time with depressive symptoms: sensitivity analysis

Variables	Sample	Depressive symptoms,^a^ %	PR (95% CI)^b^	

			Model 1^c^	Model 2^d^
Low MVPA/High ST	161	13.7	1.0 (reference)	1.0 (reference)
Low MVPA/Moderate ST	423	6.9	**0.51 (0.29–0.88)**	0.62 (0.35–1.11)
Low MVPA/Low ST	393	5.6	**0.40 (0.22–0.73)**	**0.49 (0.27–0.91)**
High MVPA/High ST	87	4.6	**0.34 (0.12–1.00)**	0.35 (0.12–1.03)
High MVPA/Moderate ST	460	4.6	**0.34 (0.19–0.63)**	**0.41 (0.22–0.76)**
High MVPA/Low ST	434	4.4	**0.32 (0.17–0.60)**	**0.39 (0.21–0.73)**

BMI, body mass index; CI, confidence interval; MVPA, moderate-to-vigorous physical activity; PR, prevalence ratio; ST, sedentary time.

The total time spent on moderate-to-vigorous PA was categorized into “high PA” (≥55.8 min/week) and “low PA” (<55.8 min/week) by the median score. ST was divided into three categories: “low ST” (<3 h/day), “moderate ST” (3 to <6 h/day), and “high ST” (≥6 h/day).

^a^Depressive symptoms were dichotomized using cut-off (scores ≥48) on the Zung Self-Rating Depression Scale.

^b^Prevalence ratios and 95% confidence intervals were estimated using Poisson regression.

^c^Adjusted for sex, age, and BMI.

^d^Adjusted for sex, age, BMI, town/city of residence, having enough sleep, current alcohol drinking, current smoking, low back pain, blood pressure, history of heart disease, and history of stroke.

## DISCUSSION

This study is the first to examine the additive effect of MVPA and ST on depressive symptoms in rural Japanese adults, and it produced two major findings. First, the sufficient MVPA and low ST category had the lowest prevalence of depressive symptoms compared with the no MVPA/high ST category. Second, the insufficient MVPA/moderate ST and insufficient MVPA/low ST groups had significantly lower prevalence of depressive symptoms than the no MVPA/high ST group.

These findings are consistent with the results of a previous study by Liao et al, in which participants with sufficient MVPA and low ST had the lowest risk of depressive symptoms compared with individuals with no MVPA and high levels of ST.^19^ However, our study was slightly different from Liao et al’s study in that the association between MVPA and depressive symptoms seemed weaker than the association between ST and depressive symptoms.^19^ Although those participants who met the PA guideline (sufficient MVPA) and had high or moderate ST tended to show fewer depressive symptoms, no significant association was found.^13^ Our results showed that the association between MVPA and depressive symptoms was weaker than that between ST and depressive symptoms. A possible reason is that the proportion of our participants who met the PA guideline (22.5%) was lower than the proportions previously reported by Liao et al and Inoue et al (54.6% and 71.3%, respectively) for Japanese participants.^19^^,^^32^ The results of the sensitivity analysis showed that depressive symptoms were significantly associated with the high MVPA/low ST category and high MVPA/moderate ST category, but not with the high MVPA/high ST category. Although the sensitivity analysis may have had low statistical power, these results supported our main results.

Rural Japanese adults who spent less time on SB (<6 h/day) were less likely to have depressive symptoms, regardless of whether they had insufficient MVPA (1 to <150 min/week). It is possible that participants with both low MVPA and low ST engaged in much light-intensity PA that did not constitute either MVPA or ST. The proportion of women in our sample (61.3%) was higher than that of men (38.7%), and previous research indicates a possible sex difference in PA level. Amagasa et al examined PA and ST using accelerometers in a community-dwelling older Japanese population.^33^ Older women with less ST engaged in more light-intensity PA and had a higher total PA time than men, although less women than men met the PA guidelines. Participants in our sample who showed less ST may also have shown greater total PA, including light-intensity PA. Total PA, which includes accumulated light-intensity PA, may be negatively associated with depressive symptoms.^12^ Alternatively, light-intensity PA itself may be associated with the likelihood of depression in women.^34^ Our measurement of PA was limited, as PA was self-reported and did not include light-intensity PA; therefore, objective measures of PA should be incorporated into future studies.

Our findings may be contributing to determine intervention strategies for the treatment or prevention of depressive symptoms. Kamada et al showed that a 5-year community-wide intervention increased PA at the population level,^35^ but it is not clear whether it is possible to prevent non-communicable diseases, such as depressive symptoms. Considering the growing body of evidence for the association between ST and non-communicable diseases, future community interventions should incorporate additional strategies to not only increase PA, but also to reduce ST.^13^

This study has several potential limitations. First, the present study had a cross-sectional design and could not explain the causal relationship between the joint association of MVPA and ST with depressive symptoms. Second, sampling took place in multiple centers from three towns/cities, and the study population were participants of an annual health examination, which could have led to selection bias. Third, MVPA and ST were measured using self-report questionnaires, which may have overestimated the time spent on MVPA and underestimated the time spent on SB because of response bias. Individuals with severe mental illness may particularly underestimate the amount of ST and overestimate their MVPA levels.^36^ Finally, we could not account for any effects of unmeasured factors that may have influenced the relationship between PA and ST and depressive symptoms, such as socioeconomic status, bereavement, physical disability, poor health, and prior depression.^9^^,^^10^^,^^37^

### Conclusion

In a joint association analysis of MVPA and ST, the combination of sufficient MVPA and low ST showed the lowest risk of depressive symptoms in rural Japanese adults. Moderate ST and low ST were significantly associated with fewer depressive symptoms, regardless of insufficient MVPA. Our results suggest that promoting PA and decreasing ST may be an effective strategy for the treatment and prevention of depressive symptoms.
